# Association between arterial stiffness and long-term efficacy of renal sympathetic denervation: 5-year results of the ASORAS study

**DOI:** 10.1007/s12928-025-01191-w

**Published:** 2025-09-23

**Authors:** Kari A. Saville, Victor J. M. Zeijen, Lida Feyz, Isabella Kardys, Marcel L. Geleijnse, Nicolas M. Van Mieghem, Melvin Lafeber, Rob J. Van Der Geest, Alexander Hirsch, Joost Daemen

**Affiliations:** 1 Department of Cardiology, Thorax Center, Erasmus University Medical Center, Room Rg-628, P.O. Box 2040, 3000 CA Rotterdam, The Netherlands; 2 Department of Internal Medicine, Erasmus University Medical Center, Rotterdam, The Netherlands; 3 Department of Radiology, Leiden University Medical Center, Leiden, The Netherlands; 4 Department of Radiology and Nuclear Medicine, Erasmus MC, Rotterdam, The Netherlands

**Keywords:** Hypertension, Vascular stiffness, Sympathectomy, Antihypertensive, Agents, Magnetic resonance imaging

## Abstract

The objective was to assess the effect of preprocedural arterial stiffness on long-term blood pressure (BP) reduction following renal sympathetic denervation (RDN). In this prospective, single-arm pilot study, patients with systolic office BP ≥ 140 mmHg and mean 24-h systolic ambulatory blood pressure (ABP) ≥ 130 mmHg despite being on a minimum of 3 antihypertensive drugs underwent radiofrequency RDN. The primary efficacy outcome was the temporal evolution of mean 24-h systolic ABP throughout 5 years post RDN. Effect modification of the primary outcome was studied for baseline magnetic resonance pulse wave velocity (MR-PWV), MR aortic distensibility (MR-AoD), ultrasound carotid femoral pulse wave velocity (CF-PWV) and clinical parameters. Analyses were performed using linear mixed-effects models to account for repeated BP measurements. A total of 30 patients were enrolled, 50% were female, mean age was 62.5 ± 10.7 years. Baseline mean 24-h ABP was 146.7/80.8 ± 13.7/12.0 despite a median of 5.0 ± 2.4 defined daily doses (DDD) of antihypertensive drugs. Baseline median MR-PWV was 6.8 [25th–75th percentile: 6.1–11.0] m/s, median MR-AoD was 1.4 × 10^–3^ mmHg^−1^ [25th–75th percentile: 0.9–1.8] and mean CF-PWV was 8.5 ± 2.1 m/s. Throughout 5 years following RDN, the change in mean 24-h systolic ABP was − 11.5 [95% CI − 17.0, − 5.9] mmHg (*p* = <0.001). MR-PWV emerged as the sole significant independent effect modifier of the change in mean 24-h systolic ABP throughout 5 years following RDN (+ 1.8 [95% CI 0.7, 2.8] mmHg per m/s per 5 years; *p* = 0.001). A higher level of preprocedural arterial stiffness, as measured using MR-PWV, was associated with a smaller BP lowering effect 5 years post-RDN.

**Graphical abstract:**

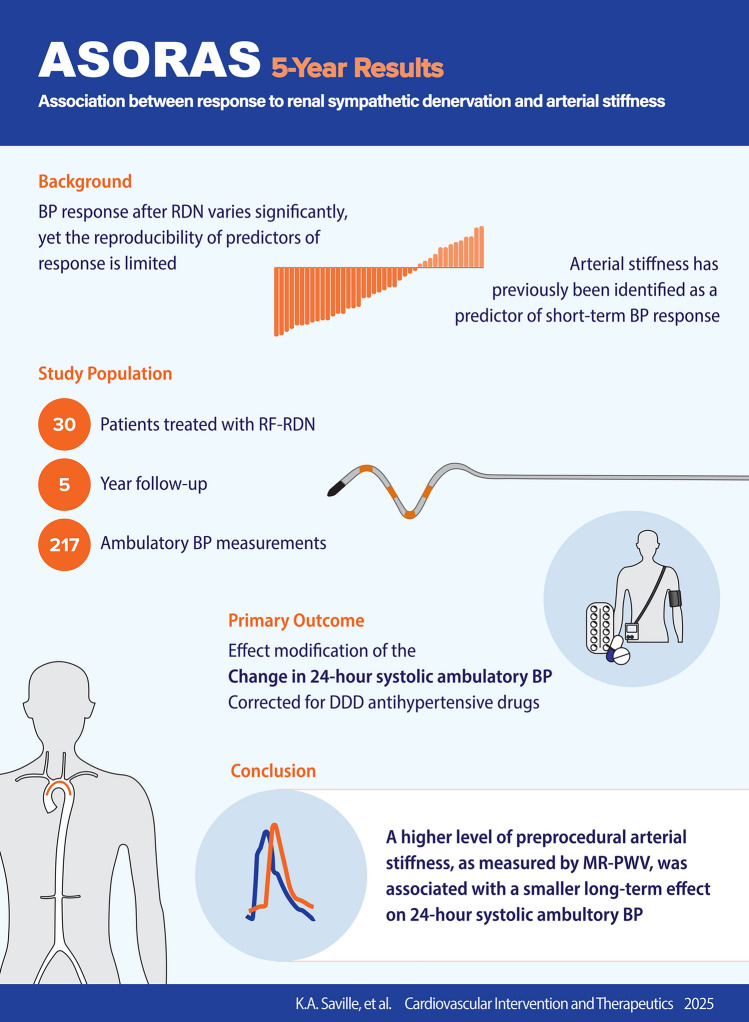

## Introduction

Hypertension is among the most common modifiable risk factors for cardiovascular disease. However, blood pressure (BP) targets are reached in merely 40% of patients [1–3]. Renal sympathetic denervation (RDN) has emerged as a novel device-based treatment option for patients with uncontrolled hypertension [4–6]. Multiple randomized controlled trials support the safety and efficacy of the treatment, showing sustained BP reductions up to at least 3 years [7]. In addition, recent registry studies have reported sustained BP reductions in parallel to a stable or reduced number of antihypertensive drugs out to 10 years after the procedure [8–10].

Nonetheless, there is a significant variability in individual patients’ response in ambulatory blood pressure reduction (ABP) post RDN [11–16]. Previous research has identified several patient characteristics and procedural characteristics as possible predictors for (non-)response, but the reproducibility of these findings across studies proved limited [17–19].

Arterial stiffness is associated with increased sympathetic nerve activity and has been identified as a prevalent and independent risk factor of cardiovascular disease [20–22]. Previous studies have found positive effects on central hemodynamics and reductions in both arterial stiffness and BP following sympathetic renal denervation, potentially lowering the risk of cardiovascular events [23, 24]. In addition, both invasively and noninvasively measured indices of arterial stiffness have been identified as predictors for the magnitude of the BP response to RDN [17, 25–28]. Specifically, invasive pulse wave velocity (PWV) has been identified as a predictor of BP response up to 6 months, magnetic resonance aortic distensibility (MR-AoD) up to 3 months, total arterial compliance up to 3 months, and carotid-femoral pulse wave velocity (CF-PWV) up to 12 months post-RDN [17, 25–28]. However, to date, no data are available on the predictive value of arterial stiffness for long-term BP reduction [25–28]. Therefore, this study aims to assess the association between noninvasive arterial stiffness and the long-term efficacy of sympathetic renal denervation.

## Materials and methods

### Study design

The current study is a predefined long-term analysis of the prospective, single-arm ASORAS study, of which the methods have been published previously [17]. In short, patients with systolic office blood pressure (OBP) ≥ 140 mmHg and mean 24-h systolic ABP ≥ 130 mmHg, despite ≥ 3 antihypertensive drugs (including at least one diuretic; or a documented intolerance to ≥ 3 classes of antihypertensive drugs) and estimated glomerular filtration rate (eGFR) ≥ 45 ml/min/1.73 m^2^ were treated with the Symplicity Flex™ single-electrode or Symplicity Spyral™ multi-electrode radiofrequency RDN system (Medtronic; Minneapolis, USA) [17]. Written informed consent was provided by all patients, the center’s local ethics committee approved the study protocol, and the study was conducted in accordance with the Declaration of Helsinki.

### Baseline examinations and follow-up

**Fig. 1:**
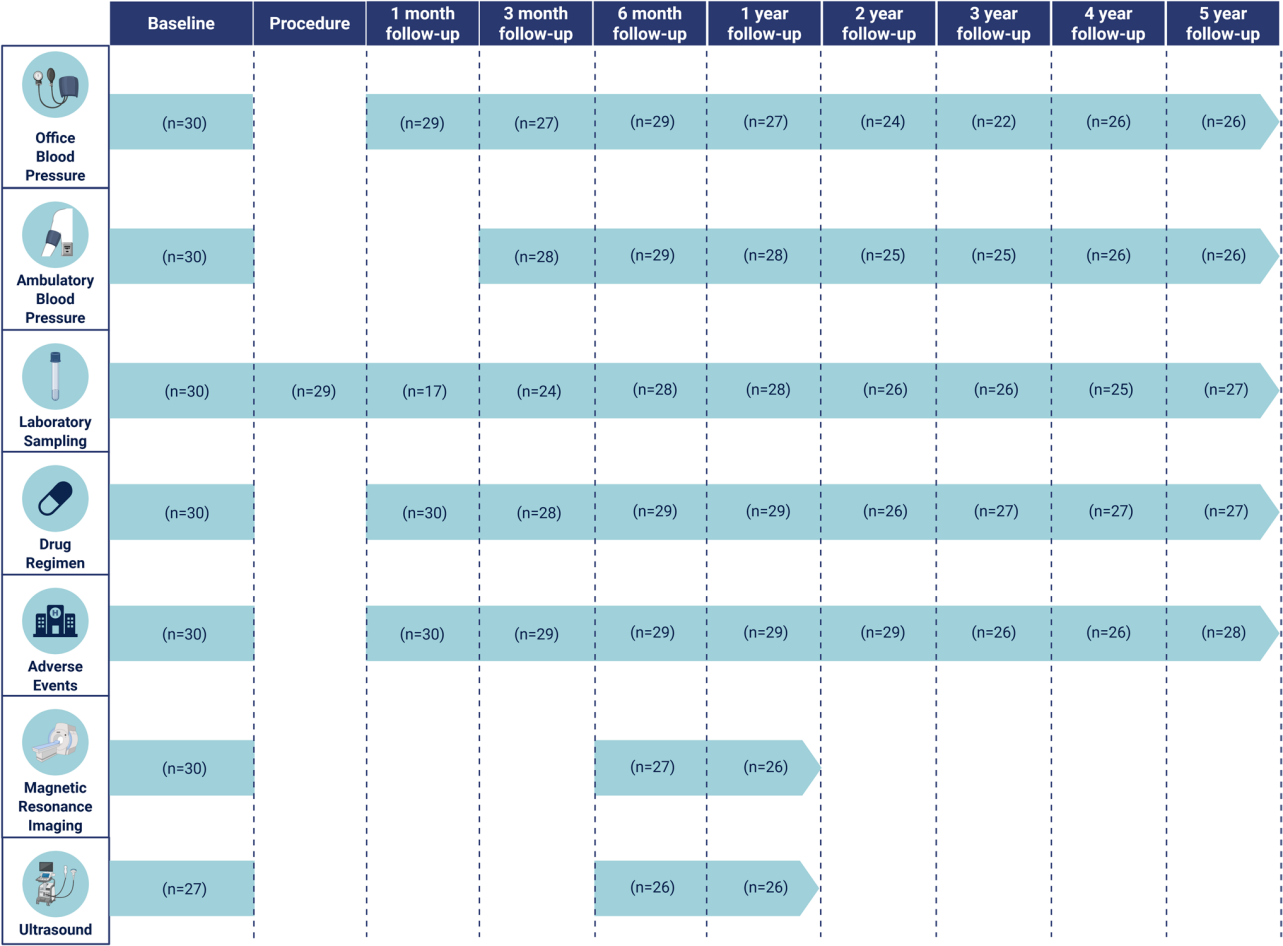
Study flowchart

Patients underwent preprocedural screening and follow-up visits at 1, 3, and 6 months and 1, 2, 3, 4, and 5 years after the procedure (Fig. 1). A physical examination, OBP measurements, and laboratory testing were performed during each visit. ABP measurement was performed at all visits except for the 1-month visit. In addition, evaluation of adverse events and antihypertensive drug regimen was performed. Defined daily doses (DDD) and the antihypertensive load index of antihypertensive drugs were calculated using the Anatomical Therapeutic Chemical classification system of the World Health Organization [29, 30]. Transthoracic echocardiography (including ultrasound CF-PWV measurement) and magnetic resonance (MR) imaging (including left ventricular (LV) volumes, mass and ejection fraction, MR-AoD and MR pulse wave velocity (MR-PWV) measurement and renal artery MR angiography) were performed at baseline, 6-month follow-up and 12-month follow-up. Methodological aspects of the BP measurements, transthoracic echocardiography and MR imaging have been published previously [17].

### Endpoints

The primary efficacy endpoint was the temporal evolution of mean 24-h systolic ABP throughout 5 years following RDN. In addition, we investigated whether this temporal evolution of 24-h systolic ABP was influenced by a predefined set of baseline covariates (effect modification), namely, arterial stiffness parameters (MR-PWV, MR-AoD, and CF-PWV), LV parameters (LV mass index and maximal LV wall thickness), transthoracic echocardiography parameters (*E*/*e* ratio, forward stroke volume index, valvulo-arterial impedance), clinical factors (age, sex, body mass index, eGFR), baseline ABP (mean 24 h, daytime, night-time), baseline OBP (including office heart rate and isolated systolic hypertension), and procedural factors (device type, number of ablations); and corrected for changes in the total number of DDD of antihypertensive drugs throughout follow-up. Secondary efficacy outcomes included the temporal evolution of other ABP outcomes (diastolic 24 h, daytime and night-time), OBP, and antihypertensive drug parameters (DDD, antihypertensive load index, number of drugs) throughout 5 years following RDN.

The primary safety endpoint was a composite endpoint consisting of cardiovascular mortality, newly acquired renal artery stenosis, the development of renal failure or requirement of dialysis, stroke, myocardial infarction, coronary revascularization and hospitalization for hypertensive emergency, whichever occurred first. Secondary safety endpoints consisted of the individual components of the composite endpoint, as well as the temporal evolution of renal function (eGFR) throughout 5 years following RDN. Renal function was calculated using the 2021 CKD–EPI formula based on serum creatinine and age at the time of measurement [31].

### Statistical analysis

Continuous variables were reported as mean ± standard deviation (SD) or median [25th–75th percentile] for normally and non-normally distributed data, respectively. Normality was assessed using the Shapiro–Wilk test and quantile–quantile plots. Categorical variables were reported as number of patients and corresponding percentages.

Linear mixed-effects models were used to analyze the temporal evolution of clinical parameters over the 5 years following RDN, with the parameter of interest as the dependent variable. Fixed effects were included for follow-up time and the number of DDD of antihypertensive drugs (time-varying covariate). Random intercepts were included to account for within-patient repeated measurements, while random slopes for time were included only if they significantly enhanced the model fit, based on the likelihood ratio test. The regression coefficients represent the change in mean 24-h systolic ABP over 5 years following RDN and are presented with the corresponding 95% confidence interval (CI) and *p* value.

Effect modification of the primary efficacy outcome was analyzed by including follow-up time, the effect modifier of interest and their interaction term [follow-up time x effect modifier] as fixed effects in the linear mixed-effects model. Subsequently, effect modifiers with a *p* value for the interaction term significant at an alpha level of 0.20 in univariable analyses were included in multivariable linear mixed-effects models. Both the univariable and multivariable mixed effect models for the change in 24-h systolic ABP were corrected for the DDD of antihypertensive drugs throughout the 5-year follow-up period. To avoid collinearity, only one measure of arterial stiffness was entered in each model, and to preserve interpretability, only one interaction term was fitted per model. The regression coefficients for the interaction terms represent the change in mean evolution of 24-h systolic ABP over 5 years following RDN, for each unit increase in continuous variables or each one-level increase in categorical variables of the baseline effect modifier. All coefficients are presented with the corresponding 95% CI and *p* value.

Safety endpoints were reported as the cumulative incidence (no. of patients and percentages) for the primary safety outcome, and as number of (repeat) events for the individual components of the composite endpoint. The temporal evolution of renal function (eGFR) throughout the 5-year follow-up period was analyzed using a similar method as for the primary efficacy outcome using a linear mixed-effects model, with eGFR as the dependent variable and a fixed effect for follow-up time.

This pilot study was considered exploratory, and no formal sample size calculation was performed to ensure detection of a predetermined effect size. Two-tailed *p* values < 0.05 were considered statistically significant unless stated otherwise. All statistical analyses were performed using R version 4.3.2, using the ‘nlme’ package for linear mixed-effects models.

## Results

### Study population and procedural characteristics

A total of 30 patients were enrolled between May 2013 and April 2019*.* Baseline characteristics are presented in Table 1**.** Five-year follow-up was completed by 26 out of 28 (92.9%) patients alive at the time of follow-up and included a total of 217 ABP measurements (Fig. 1). Fifty percent of patients were female (*n* = 15), and mean age was 62.5 ± 10.7 years*.* Baseline mean 24-h ABP was 146.7/80.8 ± 13.7/12.0 and OBP was 172.4/94.6 ± 18.7/16.0 mmHg on a regimen of 5.0 ± 2.4 DDD of antihypertensive drugs. Baseline median MR-AoD was 1.4 × 10^–3^ mmHg^−1^ [25th–75th percentile: 0.9–1.8], median MR-PWV was 6.8 [25th–75th percentile: 6.1–11.0] m/s, and mean CF-PWV was 8.5 ± 2.1 m/s. Eleven patients (37%) were treated with the Symplicity Flex™ radiofrequency RDN device, whereas 19 patients (63%) were treated with the Symplicity Spyral™ radiofrequency RDN device (Table 1).

**Table 1:** Baseline characteristics

Variable	Patients (*n* = 30)
*Clinical parameters*	
Female sex	15 (50)
Age (years)	62.5 ± 10.7
Body mass index (kg/m^2^)	29.4 ± 4.4
Body surface area (m^2^)	2.0 ± 0.3
Estimated glomerular filtration rate (ml/min/1.73 m^2^)	75.6 ± 17.0
*Smoking status*	
Current smoker	6 (20)
Ever smoker	11 (37)
*Medical history*	
Stroke and/or transient ischemic attack	3 (10)
Myocardial infarction	6 (20)
Coronary revascularization	9 (30)
Diabetes mellitus type 2	12 (40)
*Ambulatory blood pressure*	
Mean 24-h systolic blood pressure (mmHg)	146.7 ± 13.7/144.5 [137.0–152.8]
Mean 24-h diastolic blood pressure (mmHg)	80.8 ± 12.0
Daytime systolic blood pressure (mmHg)	149.8 ± 15.5/145.0 [137.3–157.5]
Daytime diastolic blood pressure (mmHg)	83.7 ± 12.8
Night-time systolic blood pressure (mmHg)	138.5 ± 14.9
Night-time diastolic blood pressure (mmHg)	74.8 ± 13.6
Ambulatory arterial stiffness index	0.53 ± 0.13
*Office blood pressure*	
Systolic blood pressure (mmHg)	172.4 ± 18.7
Diastolic blood pressure (mmHg)	94.6 ± 16.0
Heart rate (beats per minute)	67.5 [60–75.5]
Isolated systolic hypertension	13 (43)
*Antihypertensive drug treatment—summary measures*	
Defined daily doses	5.0 ± 2.4
Antihypertensive load index	2.2 [1.8–3.2]
Total number of drugs	3.4 ± 1.3
Intolerance to ≥ 3 classes of antihypertensive drugs	4 (13)
*Antihypertensive drug treatment—individual classes*	
Thiazide diuretic	23 (77)
Angiotensin converting enzyme inhibitor	5 (17)
Angiotensin receptor blocker	23 (77)
Calcium channel blocker	23 (77)
Aldosterone antagonist	6 (20)
Alpha antagonist	10 (33)
Vasodilator	4 (13)
Direct renin inhibitor	1 (3)
Cardiac magnetic resonance imaging	
LV mass index (g/m^2^)	66.8 ± 15.4
Maximal wall thickness (mm)	12.3 ± 2.7
LV end-diastolic volume index (ml/m^2^)	74.3 [69.9–86.9]
LV end-systolic volume index (ml/m^2^)	28.6 [24.0–33.1]
LV ejection fraction (%)	62.5 ± 7.7
LV stroke volume index (ml/m^2^)	48.4 ± 6.8
Cardiac output (l/min)	6.3 [5.6–7.3]
*Echocardiography*	
*E*/*e*’ ratio	14.6 ± 5.2
Forward stroke volume index (ml/m^2^)	41.2 ± 10.2
Valvulo-arterial impedance (mmHg/ml/m^2^)	4.6 ± 1.2
*Vascular parameters*	
MR-pulse wave velocity (m/s)	6.8 [6.1–11.0]
MR-aortic distensibility (10^–3^ mmHg^−1^)	1.4 [0.9–1.8]
CF-pulse wave velocity (m/s)	8.5 ± 2.1
*Procedural characteristics*	
Procedure time (minutes)	58.5 [48.5–70.0]
Contrast volume used (ml)	70.0 [50.0–120.0]
Radiofrequency renal denervation device	
Symplicity Flex™	11 (37)
Symplicity Spyral™	19 (63)
Total number of ablations bilaterally	17.5 [10.0–23.8]
Right renal emissions	6.0 [5.0–11.0]
Left renal emissions	9 [5.0–12.8]

*CF* carotid-femoral, *LV* left ventricular, *MR* magnetic resonance, *SD* standard deviation

Variables were displayed as mean ± SD, median [25th–75th percentile] or counts (percentages)

### Efficacy

**Fig. 2:**
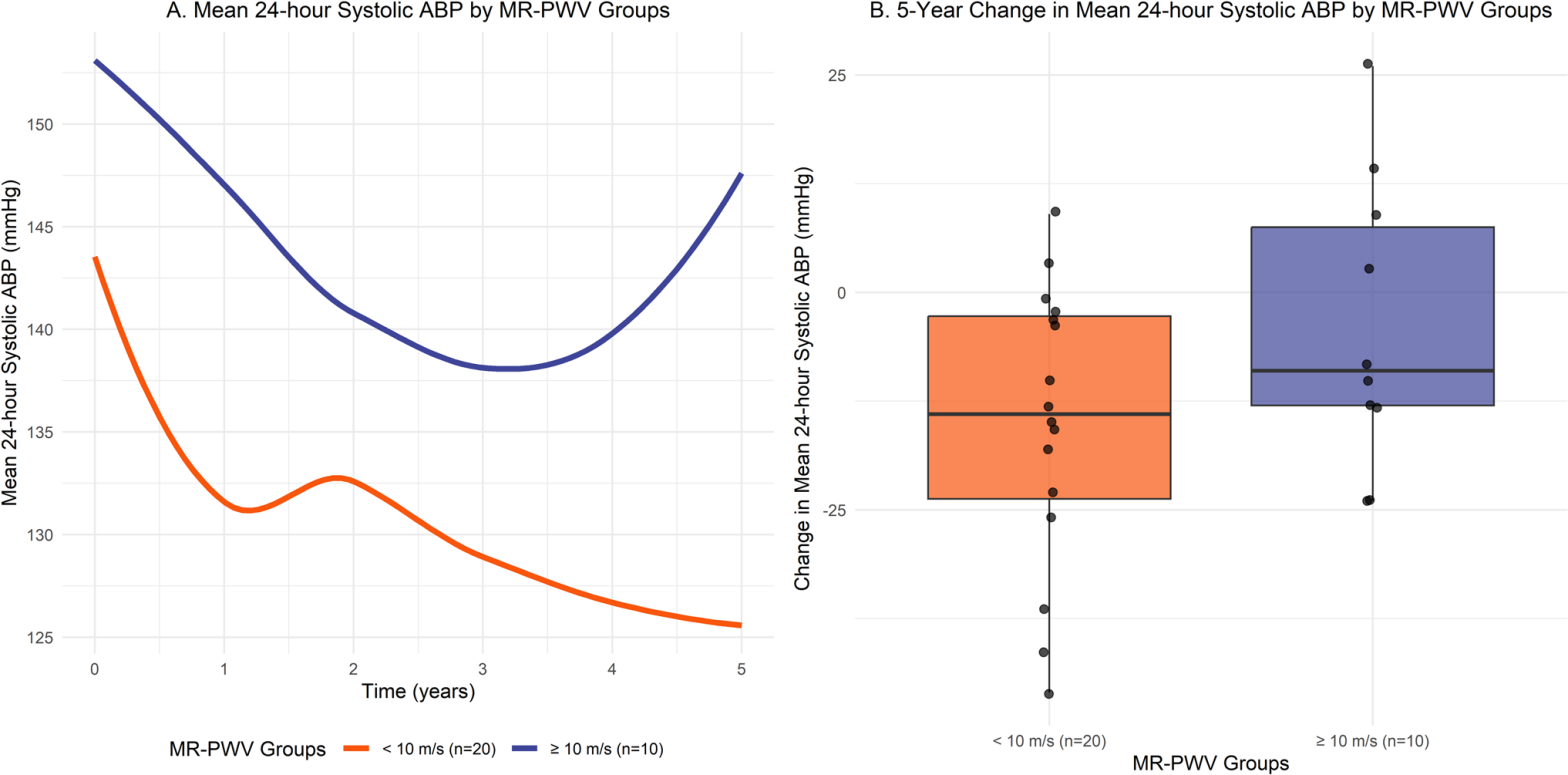
**A** Trend of mean 24-h systolic ABP by MR-PWV groups. **B** 5-year change in mean 24-h systolic ABP by MR-PWV groups

Throughout the 5-year follow-up period, a significant reduction was observed in mean 24-h systolic ABP (− 11.5 mmHg/5 years; 95% CI − 17.0, − 5.9; *p* = <0.001) (Table 2). Preprocedural MR-PWV emerged as the sole significant independent effect modifier of the change in mean evolution of 24-h systolic ABP 5 years following RDN (+ 1.8 mmHg per m/s per 5 years; 95% CI 0.7, 2.8; *p* = 0.001), corrected for DDD of antihypertensive drugs throughout follow-up, as well as baseline age, heart rate, daytime diastolic ABP and RDN device (Table 3). In addition, in patients with a higher baseline MR-PWV a trend of higher ABP was observed both preprocedural and following RDN (Fig. 2). Both preprocedural CF-PWV (+ 1.5 mmHg per m/s per 5 years; 95% CI − 0.7, 3.7; *p* = 0.18) and MR-AoD (− 3.1 mmHg per 10^–3^ mmHg^−1^ per 5 years; 95% CI − 7.4, 1.1; *p* = 0.15) did not emerge as significant independent effect modifiers for the change in mean 24-h systolic ABP throughout 5 years (Table 3). Similarly, none of the clinical or procedural characteristics demonstrated effect modification (Table 4).

**Table 2:** Change in outcome measures during 5 years after renal denervation

Variable	Modelled change post renal denervation per 5 years (95% CI)	*p* value
*Ambulatory blood pressure*		
Mean 24-h systolic blood pressure (mmHg)	− 11.5 (− 17.0, − 5.9)	<0.001
Mean 24-h diastolic blood pressure (mmHg)	− 9.0 (− 12.3, − 5.6)	< 0.001
Daytime systolic blood pressure (mmHg)	− 10.1 (− 17.0, − 3.2)	0.005
Daytime diastolic blood pressure (mmHg)	− 8.9 (− 12.7, − 5.0)	< 0.001
Night-time systolic blood pressure (mmHg)	− 8.3 (− 14.3, − 2.3)	0.007
Night-time diastolic blood pressure (mmHg)	− 6.7 (− 9.4, − 4.0)	< 0.001
*Office blood pressure*		
Systolic blood pressure (mmHg)	− 18.9 (− 24.3, − 13.5)	< 0.001
Diastolic blood pressure (mmHg)	− 9.4 (− 14.0, − 4.8)	<0.001
*Antihypertensive drugs*		
Defined daily doses	0.20 (− 0.55, 0.95)	0.59
Antihypertensive load index	− 0.09 (− 0.37, 0.18)	0.50
Total number of drugs	− 0.15 (− 0.56, 0.26)	0.47
*Renal function*		
Estimated Glomerular Filtration Rate (ml/min/1.73 m^2^)	− 11.3 (− 15.4, − 7.2)	< 0.001

**Table 3:** Multivariable analysis of baseline effect modifiers of change over time in mean 24-h systolic ambulatory blood pressure post renal denervation corrected for the DDD of antihypertensive drugs

Baseline covariates	Change in mean 24-h systolic ABP post renal denervation in mmHg/5 years (95% CI)^a^	*p* value
*Vascular parameters*		
MR-pulse wave velocity (m/s)	1.8 (0.7, 2.8)	0.001
MR-aortic distensibility (10^–3^ mmHg^−1^)	− 3.1 (− 7.4, 1.1)	0.15
CF-pulse wave velocity (m/s)	1.5 (− 0.7, 3.7)	0.18
*Clinical parameters*		
Age (years)	0.3 (− 0.1, 0.7)	0.11
*Office blood pressure*		
Heart rate (beats per minute)	− 0.2 (− 0.4, 0.05)	0.14
*Ambulatory blood pressure*		
Daytime diastolic blood pressure (mmHg)	− 0.3 (− 0.6, 0.1)	0.19
*Procedural characteristics*		
Spyral™ device (as compared to Flex™ device)	6.8 (− 2.5, 16.1)	0.15

^a^Multivariable models were fitted with only one interaction term per model to preserve interpretability and one measure of arterial stiffness to avoid collinearity. All models were corrected for the baseline values of the covariates in Table 3 and the DDD of antihypertensive drugs

^b^The regression coefficients for the interaction terms represent the change in mean evolution of 24-h systolic ABP over 5 years following RDN, for each unit increase in continuous variables or each one-level increase in categorical variables of the baseline effect modifier. For example, an increase in MR-pulse wave velocity of 1 m/s at baseline corresponds with an 1.8 mmHg increase in mean 24-h systolic ABP at 5 years

**Table 4:** Univariable effect modifiers of change in mean 24-h ambulatory systolic blood pressure post renal denervation corrected for DDD antihypertensive medication

Baseline covariates	Change in mean 24-h systolic ABP post renal denervation in mmHg/5 years (95% CI)^a^	*p* value
*Clinical parameters*		
Age (years)	0.3 (− 0.1, 0.7)	0.11
Female sex (as compared to male)	1.4 (− 7.5, 10.5)	0.75
Body Mass Index (kg/m^2^)	− 0.2 (− 1.2, 0.9)	0.73
Estimated Glomerular Filtration Rate (ml/min/1.73 m^2^)	− 0.1 (− 0.4, 0.2)	0.38
*Ambulatory blood pressure*		
Mean 24-h systolic blood pressure (mmHg)	− 0.05 (− 0.5, 0.4)	0.83
Mean 24-h diastolic blood pressure (mmHg)	− 0.3 (− 0.7, 0.2)	0.23
Daytime systolic blood pressure (mmHg)	− 0.09 (− 0.5, 0.3)	0.64
Daytime diastolic blood pressure (mmHg)	− 0.3 (− 0.6, 0.1)	0.16
Night-time systolic blood pressure (mmHg)	0.2 (− 0.2, 0.5)	0.34
Night-time diastolic blood pressure (mmHg)	− 0.06 (− 0.4, 0.3)	0.74
Ambulatory arterial stiffness index	20.8 (− 15.4, 57.0)	0.26
*Office blood pressure*		
Systolic blood pressure (mmHg)	0.1 (− 0.1, 0.4)	0.25
Diastolic blood pressure (mmHg)	0.1 (− 0.2, 0.4)	0.65
Heart rate (beats per minute)	− 0.2 (− 0.4, 0.0)	0.12
Isolated systolic hypertension	− 4.8 (− 13.6, 4.1)	0.29
*Cardiovascular magnetic resonance*		
LV mass index (g/m^2^)	− 0.1 (− 0.5, 0.2)	0.52
Maximal wall thickness (mm)	− 0.4 (− 2.5, 1.6)	0.68
*Echocardiography*		
*E*/*e*’ ratio	− 0.3 (− 1.2, 0.7)	0.58
Forward stroke volume index (ml/m^2^)	0.2 (− 0.2, 0.7)	0.32
Valvulo-arterial impedance (mmHg/ml/m^2^)	− 1.4 (− 5.1, 2.2)	0.44
*Vascular parameters*		
MR-pulse wave velocity (m/s)	1.8 (0.7, 2.8)	0.001
MR-aortic distensibility (10^–3^ mmHg^−1^)	− 3.1 (− 7.4, 1.2)	0.15
CF-pulse wave velocity (m/s)	1.4 (− 0.8, 3.7)	0.20
*Procedural characteristics*		
Spyral™ device (as compared to Flex™ device)	6.9 (− 2.4, 16.2)	0.15
Total number of ablations	0.2 (− 0.3, 0.8)	0.39

^a^The regression coefficients for the interaction terms represent the change in mean evolution of 24-h systolic ABP over 5 years following RDN, for each unit increase in continuous variables or each one-level increase in categorical variables of the baseline effect modifier. For example, an increase in MR-pulse wave velocity of 1 m/s at baseline corresponds with an 1.8 mmHg increase in mean 24-h systolic ABP at 5 years. All univariable models were corrected for the DDD of antihypertensive drugs

Significant reductions in BP throughout the 5-year follow-up period were observed across all BP measures (Table 2). Throughout follow-up, the number of DDD of antihypertensive drugs remained stable at 0.20 per 5 years (95% CI 0.55, 0.95; *p* = 0.59).

### Safety

The primary safety endpoint occurred in 12 patients (40%). Detailed information on the long-term clinical follow-up are presented in Table 5. One patient (3%) developed a renal artery stenosis 4 years post-RDN. Renal function (eGFR) changed with − 11.3 ml/min/1.73 m^2^/5 years (95% CI − 15.4, − 7.2, *p* < 0.001).

**Table 5:** Safety endpoints at 5 years

Clinical endpoint	Number of patients (%)
Primary safety endpoint (cardiovascular mortality, renal artery stenosis, renal failure, stroke, myocardial infarction, hospitalization for hypertensive emergency, coronary revascularization)	12 (40%)

	Number of events
Cardiovascular mortality	2
Newly acquired renal artery stenosis and/or repeat renal artery intervention	1
Development of renal failure or requirement of dialysis	0
Stroke or transient ischemic attack	4
Myocardial infarction	1
Hospitalization for hypertensive emergency	6
Coronary revascularization	4

## Discussion

This single-center pilot study aimed to assess the association between preprocedural arterial stiffness and the long-term change in BP following RDN. Baseline MR-PWV was identified as the sole independent effect modifier of the reduction in mean 24-h systolic ABP following RDN.

Throughout the 5-year follow-up period, we observed a significant reduction in mean 24-h systolic ABP of − 11.5 mmHg. With the intention to assess the potential predictive value of arterial stiffness on the long-term BP lowering effect of RDN, baseline MR-PWV emerged as the only significant independent effect modifier, with higher baseline values resulting in a smaller decrease in mean 24-h systolic ABP throughout the 5-year period (+ 1.8 mmHg per m/s per 5 years). In contrast, neither CF-PWV (+ 1.5 mmHg per m/s per 5 years), MR-AoD (− 3.1 mmHg per 10^–3^ mmHg^−1^ per 5 years), nor any of the assessed clinical or procedural characteristics were found to be independent effect modifiers (Table 4).

Stiffening of the large arteries is a key determinant of isolated systolic hypertension and the age-dependent increase in pulse pressure [32]. Longstanding hypertension further contributes to increased vascular stiffness by increasing pulsatile aortic wall stress, creating a perpetuating feedback loop [33, 34]. This was also reflected in the findings of the current study, as higher baseline ABP values were observed in patients with higher baseline MR-PWV values. Moreover, both hypertension and arterial stiffness have been independently associated with an increase in sympathetic nerve activity [20–22, 35].

Notably, previous studies have also shown that elevated baseline pulse wave velocity is associated with smaller blood pressure reductions in response to pharmacologic therapy, suggesting that increased arterial stiffness may serve as a general indicator of reduced responsiveness to antihypertensive treatment [36, 37].

Though CF-PWV measurements are easy to implement in clinical practice, MR-PWV provides more accurate aortic distance measures, especially in patients with central obesity [38, 39]. In addition, in patients with hypertension, the aorta stiffens more than the carotid artery with age and other cardiovascular risk factors, possibly leading to an underestimation of true arterial stiffness using CF-PWV [40]. While routine use of arterial stiffness to guide patient selection is not currently established, our findings raise the hypothesis that MR-PWV may prove useful in this regard. As MR imaging is often part of the preprocedural renal artery anatomy assessment, incorporating MR-PWV into future studies or registries could help validate its role. In clinical practice, more accessible methods such as CF-PWV may serve as practical alternatives. Larger studies are needed to define diagnostic accuracy of pragmatic cutoff values to identify potential responders.

Both invasive and noninvasive measures of arterial stiffness have been previously studied as predictors of response to RDN [25–27]. However, these studies evaluated only short-term effects on BP out to 12 months. The current study is the first to assess both the short- and long-term effect on BP reduction over a 5-year follow-up period. In addition, several factors should be considered when defining a response to RDN to accurately identify predictors [19]. Using linear mixed-effects models, our analysis approach accounted for multiple repeated measurements, incorporated visit-to-visit BP variation, and increased statistical power based on 217 observations. In addition, our models accounted for BP variation over time within and between individuals. In parallel, models were corrected for changes in patients’ concomitant antihypertensive drug regimens throughout follow-up, to rule out the effect of changes in drug burden on the relationship between preprocedural arterial stiffness and 5-year BP outcomes.

Unfortunately, the reproducibility of the various noninvasive measures as predictors for BP response after RDN has been limited [17, 25, 28]. The latter is likely driven by the limited sample size of most previous studies, dichotomization of BP response and a wide variety of methods used for the assessment of arterial stiffness, thereby precluding any comparisons of our findings to previous work.

The long-term follow-up of the present study again illustrates the significant burden of disease associated with uncontrolled BP with 12 patients experiencing a major cardiovascular event throughout the 5-year follow-up (40%), reflecting a high-risk population and supporting the recent adoption of RDN in clinical practice guidelines as an adjunct treatment option for uncontrolled hypertension [4, 6].

We observed a significant 5-year change in renal function (eGFR) of -11.3 ml/min/1.73 m^2^/5 years, equal to a decline of 2.3 ml/min/1.73 m^2^/year. Previous studies have reported reductions in eGFR ranging from 0.9 to 2.1 ml/min/1.73 m^2^/year, similar to the natural decline in renal function in patients with uncontrolled hypertension [41]. The numerically higher reduction in eGFR in the present pilot study can be explained by the high-risk entity of our study population at baseline, together with one patient who developed a renal artery stenosis leading to a clinically significant reduction in renal function [42].

## Limitations

This study has several limitations. First, the current study was a pilot study, and therefore, no formal sample size calculation was performed to ensure detection of a predetermined effect size. The latter could have resulted in false-negative results (type II error). Second, the absence of a (sham-) control group precludes any causal statements regarding the effect of RDN on BP over time as well as the modifying effect of baseline arterial stiffness. To overcome these limitations, adequately powered randomized studies will be required to provide more insights on the effect of arterial stiffness on RDN-mediated BP reduction. Third, we corrected for prescribed antihypertensive drug DDDs in our primary efficacy outcome, but we cannot rule out a change in patient adherence over time as this was not routinely measured. Fourth, vascular parameters were only measured until 12months post-RDN, and therefore, the long-term effect of RDN on vascular stiffness could not be assessed. Finally, 11 out of 30 patients were treated with the Simplicity Flex catheter, a device that is no longer commercially available.

## Conclusion

Arterial stiffness, as measured using MR-PWV, was an independent effect modifier of the reduction in BP throughout 5 years following RDN, with higher levels of arterial stiffness corresponding with a smaller decrease in ABP after RDN.

## Data Availability

The data that support the findings of this study are available from the corresponding author upon reasonable request.
